# Estimation of Genetic Parameters for Carcass and Meat Quality Traits Using Genomic Information in Yorkshire Pigs

**DOI:** 10.3390/ani15142075

**Published:** 2025-07-14

**Authors:** Yangxun Zheng, Fuping Ma, Xitong Zhao, Yanling Liu, Quan Zou, Huatao Liu, Shujuan Li, Zipeng Zhang, Sen Yang, Kai Xing, Chuduan Wang, Xiangdong Ding

**Affiliations:** 1 State Key Laboratory of Animal Biotech Breeding, National Engineering Laboratory for Animal Breeding, Key Laboratory of Animal Genetics, Breeding and Reproduction of Ministry of Agriculture and Rural Affairs, College of Animal Science and Technology, China Agricultural University, Beijing 100193, China; yxzheng314@163.com (Y.Z.); ma_fuping@163.com (F.M.); liuyanl2022@163.com (Y.L.); s20193040533@cau.edu.cn (Q.Z.); liuhuatao@cau.edu.cn (H.L.); sjuan2021@163.com (S.L.); zzp13226665505@126.com (Z.Z.); xk995@126.com (K.X.); 2 Beijing Shunxin Agriculture Co., Ltd., Beijing 101300, China; zhaoxitong0348@163.com (X.Z.); djz1300@163.com (S.Y.)

**Keywords:** carcass traits, meat quality, phenotypic correlation, genetic correlation, heritability, Yorkshire

## Abstract

Carcass and meat quality traits are critical in pig breeding and production. Estimating genetic parameters for these traits is a vital aspect of breeding engineering, as accurate genetic parameters are essential for estimating breeding values, predicting genetic progress, and optimizing breeding programs. This study was conducted on a population of 461 Yorkshire pigs from the same breeding farm, which were slaughtered to assess nine carcass traits and seven meat quality traits, followed by descriptive statistical analysis. Additionally, we estimated the genetic parameters of these traits using genomic information based on 50K chip data. The results indicated that sex significantly affected most carcass and meat quality traits. Carcass traits including carcass length indicators (h^2^ = mean 0.35), backfat thickness indicators (h^2^ = mean 0.36), eye muscle area (h^2^ = 0.28), and the number of rib pairs (h^2^ = 0.28) exhibited medium to high heritability. Carcass length indicators showed high genetic correlations with backfat thickness indicators (r = mean −0.49) and the number of rib pairs (r = mean 0.63), while high negative genetic correlation (r = −0.72) was noted between eye muscle area and the number of rib pairs. Meat quality traits also displayed medium to high heritability, expect for pH value measured within one hour post-slaughter (h^2^ = 0.12). Drip loss indicators had higher genetic correlations with pH (r = mean −0.73) than with meat color indicators (r = mean 0.22). These findings may provide a theoretical reference for genetic evaluation and breeding in the Yorkshire pig population.

## Simple Summary

A total of 461 Yorkshire pigs were slaughtered in this study. Descriptive statistical analyses were performed on nine carcass traits and seven meat quality traits. The genetic parameters of these traits were estimated using genomic information. The results indicated that the gender of Yorkshire pigs significantly influenced most carcass traits and meat quality traits. All carcass traits and most meat quality traits exhibited medium to high heritability. The carcass length indicators demonstrated substantial genetic correlations with both backfat thickness indicators and the number of rib pairs. Conversely, a pronounced negative genetic correlation was observed between the eye muscle area and the number of rib pairs. Additionally, the genetic correlations of drip loss indicators were higher with pH values than with meat color indicators. These findings provide a theoretical reference for promoting genetic progress and optimizing breeding programs in Yorkshire pig populations.

## 1. Introduction

Carcass traits (e.g., carcass length, backfat thickness, eye muscle area, and number of rib pairs) are important economic traits in pig production and breeding. Carcass traits can intuitively reflect growth development and are closely related to physiological function, production performance, disease resistance, and environmental adaptability [1]. Carcass length, as the most direct production indicator in the pig industry, is one of the most important phenotypic indexes reflecting the overall appearance [2]. A study on the genetic parameters of these external traits is helpful to accelerate the genetic improvement of related traits. Backfat, the subcutaneous adipose tissue spanning from the dermal layer to the longissimus dorsi, serves as an important evaluation index of carcass lean percentage and sow reproductive performance [3]. Backfat thickness exhibits correlations with meat quality traits including intramuscular fat content, juiciness, and flavor [4]. The eye muscle area, defined as the cross-sectional area of the longissimus dorsi, correlates with carcass leanness and meat yield. Thoracic vertebral counts determine the number of rib pairs (14 to 17), and directly impact carcass length. King et al. [5] revealed that each vertebrae contributed approximately 15 mm to carcass length.

Consumers’ perception of pork eating quality are primarily driven by sensory scores for juiciness and tenderness, which are associated with shear force, cooking loss, and final pH [6,7]. Meat quality indicators primarily comprise intramuscular fat content, drip loss, pH value, and meat color. Intramuscular fat mainly presents in the muscle fibers and muscle fiber bundles, and its content, as an essential factor affecting meat quality, is closely related to color, tenderness, juiciness, and flavor [8]. Drip loss directly indicates the water-holding capacity of meat. A greater drip loss indicates that more water is excluded from the meat, resulting in poorer juiciness, flavor, and texture of the meat. The pH value is a crucial indicator of meat quality, with post-slaughter pH fluctuations significantly impacting the muscle’s water-holding capacity [9]. Parameters such as meat color, texture, and muscle pH at various post-slaughter intervals are frequently referenced as criteria for assessing whether meat is physiologically normal or abnormal. The initial consumer perception of meat quality is largely influenced by its color [10], which is primarily determined by the presence of myoglobin, hemoglobin, cytochromes, and other pigmented compounds within the muscle. Abnormal meat conditions, such as dark, firm, and dry (DFD) or pale, soft, and exudative (PSE) meat, are characterized by atypical dark or pale coloration.

Accurate genetic parameters are vital for estimating breeding values, predicting genetic progress, and optimizing breeding programs. The estimation of genetic parameters for economically significant traits (carcass and meat quality) in pigs is a crucial aspect of breeding engineering. With the advancement of sequencing technologies, the estimation of genetic parameters has shifted from being based on pedigree information to genomic information. Genomic information more accurately reflects the relationships between individuals than pedigree information [11], thereby improving the accuracy of genetic parameter estimation. In this study, a cohort of Yorkshire pigs was utilized as the research subject to assess 16 indicators (thoracolumbar length, carcass length, carcass straight length, carcass slanting length, backfat A, backfat B, backfat C, number of rib pairs, the eye muscle area, intramuscular fat content, drip loss at 24 or 48 h, pH, lightness, redness, and yellowness) related to carcass traits and meat quality traits post-slaughter. Each heritability for these 16 indicators was estimated based on genomic information, and the study analyzed the various influencing factors on carcass and meat quality traits as well as the intraclass phenotypic and genetic correlations in carcass indicators or meat quality indicators. The findings of this study provide a foundation for enhancing the accuracy of breeding and developing rational breeding programs.

## 2. Materials and Methods

### 2.1. Phenotypic and Genetic Data

#### 2.1.1. Animals and Phenotype

The experimental pigs were sourced from a pig breeding farm of Beijing Shunxin Agricultural Co., Ltd. (Beijing, China). All pigs were maintained under identical feeding conditions until approximately 180 days of age. A total of 461 Yorkshire pigs (289 boars and 172 sows) with complete individual information and phenotypic records were included in subsequent analyses. The nomenclature, definitions, and abbreviations of carcass and meat quality traits are presented in Table 1. Carcass length indicators, including the length from the first thoracic vertebra to the last lumbar vertebra (CL1), the length from the anterior edge of the pubic symphysis to the anterior edge of the first cervical spine (CL2), the length from the lumbosacral junction to the anterior edge of the first cervical spine (CL3), and the length from the anterior edge of the pubic symphysis to the junction of the first rib and sternum (CL4), were measured with a soft tape measure. Backfat thickness indicators, including the backfat thickness in the withers (BFA), thoracolumbar junction (BFB), and lumbar spine junction (BFC), were measured with an electronic vernier caliper. Number of rib pairs (NR) was recorded. The contours of the eye muscles were drawn on sulfuric acid paper to measure eye muscle area (EMA) using a planimeter (Koizumi Placom, KP-90N, Niigata, Japan). Meat quality traits included intramuscular fat content (IMF), pH value measured within one hour after slaughter (pH_1_), drip loss at 24 h and 48 h (DL24, DL48), and meat color value indicators (L*, a*, b*). Details of materials and procedures can be found in previous publication from our group [12].

#### 2.1.2. Genotyping and Quality Control

Longissimus dorsi muscle tissue was collected from all pigs, and genomic DNA was extracted using a high-throughput DNA extraction kit. DNA quality was assessed through two methods: (1) purity and integrity analysis via 1% agarose gel electrophoresis and (2) quantification using a Qubit Fluorometer. Qualified DNA samples were normalized to 50 ng/µL and genotyped using the GenoBaits Porcine SNP50K Panel (MolBreeding Biotechnology Co., Ltd., Shijiazhuang, China). This array contains 52,000 genome-wide SNPs.

Genetic data quality control was performed with PLINK software (v1.90) [13] under the following criteria: (1) exclusion of SNPs located on sex chromosomes; (2) removal of SNPs with call rates < 95%; (3) elimination of SNPs with minor allele frequency (MAF) < 1%; (4) exclusion of SNPs deviating from Hardy-Weinberg equilibrium (*p* < 1 × 10^−6^); and (5) removal of individuals with call rates < 90%. After quality control, 460 individuals and 45,112 SNPs were retained for downstream analyses. This is in accordance with previous work in our group [12].

### 2.2. Methods

#### 2.2.1. Descriptive Statistics, Normality Test, and Phenotypic Correlation Analysis

Descriptive statistics, including mean, standard deviation, coefficient of variation, and maximum and minimum values, were calculated for carcass and meat quality traits by R software (v 4.1.3). The normality of phenotypic values was assessed through the Shapiro-Wilk test [14]. Pearson correlation coefficients were calculated to evaluate phenotypic associations among traits.

#### 2.2.2. Fixed Effects Classification and Significance Test

Based on the experimental conditions, the following fixed effects were considered in genetic parameter estimation for Yorkshire pigs: slaughter batch (five levels based on slaughter dates), sex (boar/sow), and evaluator (six levels corresponding to measurement operators), with carcass weight included as a covariate. Generalized Linear Models were employed to assess fixed effect significance using the following model:

(1) $$Y_{ijkh}=\mu +B_{i}+S_{j}+M_{k}+aW_{h}+e_{ijkh},$$

where Y_ijkh_ represents the phenotypic value; μ is the population mean; B_i_ represents the batch effect (i = 1 to 5); S_j_ indicates the sex effect (j = 1, 2); M_k_ corresponds to the evaluator effect (k = 1 to 6); W_h_ represents the carcass weight of the h-th individual; e_ijkh_ stands for the random residual error; and a represents the regression coefficient for the carcass weight. Notably, EMA was measured using a planimeter, minimizing human intervention. Consequently, the evaluator effect was excluded for this trait. Moreover, as NR showed no association with carcass weight, this trait’s analysis omitted carcass weight as a covariate. For meat quality traits, solely batch and sex effects were included in the statistical model.

#### 2.2.3. Estimation of Genetic Parameters

Genetic variance explained by SNPs was estimated using the Genome-based Restricted Maximum Likelihood (GREML) method [15,16] implemented in GCTA (v1.94.0) [17]. The bivariate GREML method was applied to estimate genetic correlations among traits [18]. The statistical analysis models are as follows:

1. Model for estimating heritability:

(2) y=Xβ+Zg+ɛ,

where y is a vector of phenotypic values; β is a vector including covariate and significant fixed effects; g is a vector of all autosomal SNP effects, where $var(g)=A\sigma_{g}^{2}$ and A is a genetic relationship matrix; X and Z are design matrices; and ɛ is a vector of random residual effect. The heritability was calculated by $h_{g}^{2}=\sigma_{g}^{2}/\sigma_{p}^{2}$, in which σ_g_^2^ is the genetic variance and σ_p_^2^ is the phenotypic variance.

2. The bivariate GREML model:

(3) $$\left[\begin{array}{c} y_{1}\\ y_{2} \end{array}\right]=\left[\begin{array}{c} X_{1}\beta_{1}\\ X_{2}\beta_{2} \end{array}\right]+\left[\begin{array}{c} {Z_{1}g}_{1}\\ {Z_{2}g}_{2} \end{array}\right]+\left[\begin{array}{c} {ɛ}_{1}\\ {ɛ}_{2} \end{array}\right]$$

The variance covariance matrix (V) between $y_{1}$ and $y_{2}$ is defined as:

(4) $$V=\left[\begin{array}{cc} Z_{1}AZ_{1}^{\prime }\sigma_{g_{1}}^{2}+I\sigma_{\varepsilon_{1}}^{2} & Z_{1}AZ_{2}^{\prime }\sigma_{g_{1}g_{2}}^{2}\\ Z_{2}AZ_{1}^{\prime }\sigma_{g_{1}g_{2}}^{2} & Z_{2}AZ_{2}^{\prime }\sigma_{g_{2}}^{2}+I\sigma_{\varepsilon_{2}}^{2} \end{array}\right]$$

where these symbols are defined in the same way as the model used to estimate heritability, and subscripts 1 and 2 represent trait 1 and trait 2. For example, $y_{1}$ and $y_{2}$ are two vectors of phenotypic values for trait 1 and trait 2. I is the unit matrix, and $\sigma_{g_{1}g_{2}}^{2}$ is the covariance between $g_{1}$ and $g_{2}$.

## 3. Results

### 3.1. Descriptive Statistics of Carcass Traits and Meat Quality Traits

The descriptive statistics and normality tests for carcass and meat quality traits are presented in Table 2 and Table 3. In carcass traits, backfat thickness indicators exhibited the highest coefficients of variation, while the carcass length indicators showed the lowest (Table 2). Shapiro-Wilk tests revealed that CL2, CL4, BFA, and BFB followed a normal distribution (*p* > 0.05) in carcass traits, whereas the remaining traits deviated from normality (*p* ≤ 0.05) (Table 2). For meat quality traits, substantial variation was observed in IMF, DL24, DL48, and b*, while variations in pH_1_, L*, and a* were within a single digit percentage range (Table 3). Only a* followed a normal distribution (*p* > 0.05), with all others showing significant deviations (*p* ≤ 0.05) (Table 3).

**Figure 1:**
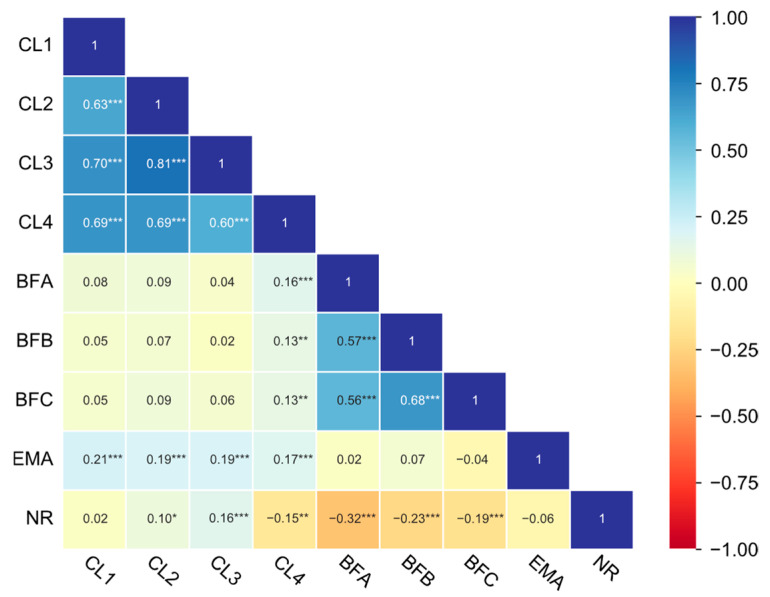
Phenotypic correlation among carcass traits (*** *p* < 0.001, ** *p* < 0.01, and * *p* < 0.05).

**Figure 2:**
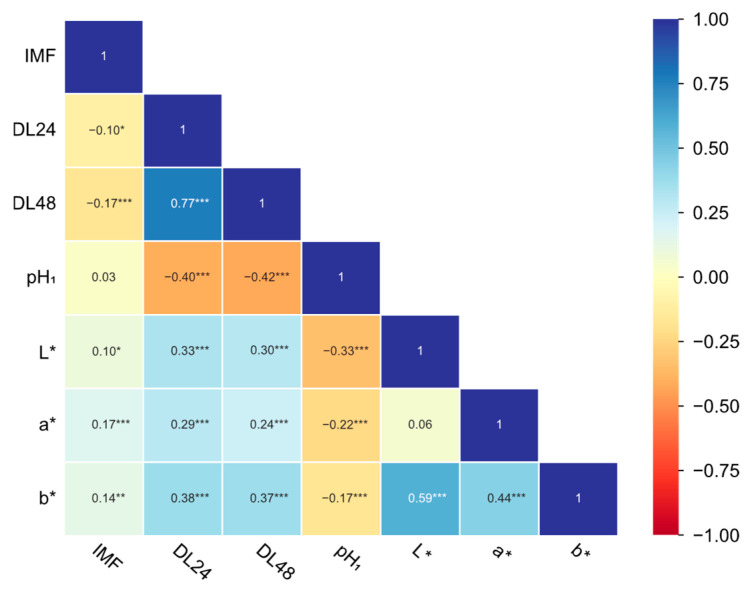
Phenotypic correlation among meat quality traits (*** *p* < 0.001, ** *p* < 0.01, and * *p* < 0.05). Note: This was reported in our previous work [12].

The phenotypic correlation matrix revealed distinct association patterns among carcass traits (Figure 1). Strong and significant (*p* < 0.001) positive correlations were observed within trait categories: carcass length indicators (r = 0.60 to 0.81) and backfat thickness indicators (r = 0.56 to 0.68). Carcass length indicators and backfat thickness indicators showed weak positive correlations (r = 0.02 to 0.16). EMA was significantly (*p* < 0.001) positively correlated with carcass length indicators, whereas the NR exhibited significantly (*p* < 0.001) inverse relationships with backfat thickness indicators. The phenotypic correlation matrix among meat quality traits is illustrated in Figure 2. IMF exhibited weak associations (r = −0.17 to 0.17) with other traits, while DL24 and DL48 demonstrated the strongest significant correlation (r = 0.77, *p* < 0.001). Drip loss indicators (DL24/DL48) and pH_1_ indicated moderately strong negative associations (r = −0.40 and −0.42, *p* < 0.001). pH_1_ displayed significant (*p* < 0.001) weak negative correlations with meat color indicators lightness (L*: r = −0.33), redness (a*: r = −0.22), and yellowness (b*: r = −0.17), while drip loss indicators displayed significant (*p* < 0.001) weak positive correlations with meat color indicators. Notably, meat color indicators demonstrated intercorrelations, particularly between yellowness and lightness (r = 0.59) or redness (r = 0.44).

The fixed-effects analysis identified significant influencing factors on carcass and meat quality traits (Table 4). The impact of sex was significant (*p* < 0.05), moderately significant (*p* < 0.01), or extremely significant (*p* < 0.001) for most traits. For carcass traits, the batch only had a significant impact on CL3, BFC, and EMA. The evaluator showed extreme significance for CL1, CL3, BFC, and NR, with moderate significance observed for CL2, CL4, and BFA. For meat quality traits, the impact of batch was extremely significant for pH_1_ and b*.

### 3.2. Carcass Traits’ Heritability and Genetic Correlations

**Figure 3:**
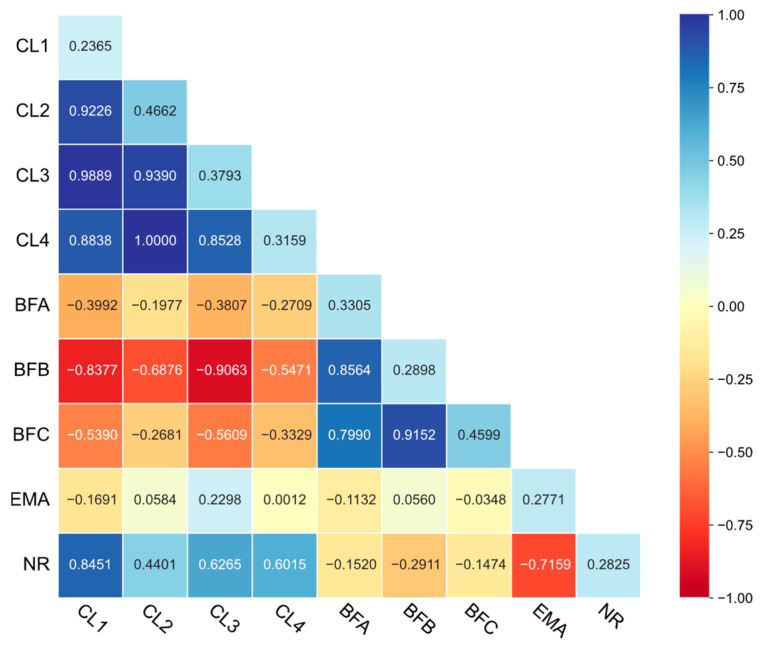
Genomic genetic correlation among carcass traits. Genomic heritability of each carcass trait is on the diagonal.

The genomic heritability estimates for carcass length indicators ranged from 0.24 to 0.47, while those for backfat thickness indicators ranged from 0.29 to 0.46 (Figure 3). EMA and NR exhibited a heritability estimate of 0.28. Specifically, CL2 and BFC demonstrated high heritability (h^2^ > 0.4), whereas the remaining traits exhibited moderate heritability (0.2 ≤ h^2^ ≤ 0.4).

Strong intraclass genetic correlations emerged, with carcass length indicators showing r = 0.85 to 1.00 and backfat thickness indicators showing r = 0.80 to 0.92 (Figure 3). High negative genetic correlations were observed between carcass length indicators and backfat thickness indicators, peaking at r = −0.91 (BFB and CL3). EMA displayed weak genetic associations with other traits (r = −0.17 to 0.23). NR showed a high positive genetic association with carcass length indicators (r = 0.44 to 0.85) while exhibiting moderate negative correlations with backfat thickness indicators (r = −0.29 to −0.15) and a high inverse relationship with EMA (r = −0.72).

### 3.3. Meat Quality Traits’ Heritability and Genetic Correlations

**Figure 4:**
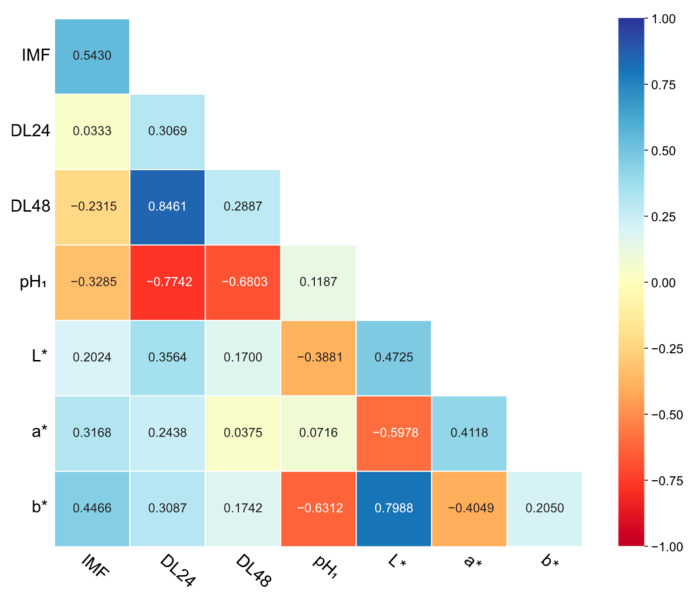
Genomic genetic correlation among meat quality traits. Genomic heritability of each meat quality trait is on the diagonal.

Meat quality traits exhibited low to high genomic heritability (h^2^ = 0.12 to 0.54), as depicted in Figure 4. pH_1_ was classified as a low heritability (h^2^ < 0.2) trait, primarily governed by environmental factors. DL24, DL48, and b* exhibited moderate heritability (0.2 ≤ h^2^ ≤ 0.4). IMF, L*, and a* showed high heritability (h^2^ > 0.4), indicating strong genetic control, thus enabling effective genetic improvement. Notably, IMF had the maximum heritability estimate (h^2^ = 0.54).

The highest genetic correlation was found between DL24 and DL48 (r = 0.85), whereas the lowest genetic correlation occurred between IMF and DL24 (r = 0.03) (Figure 4). IMF displayed moderate genetic correlations (r = −0.33 to 0.45) with other traits. pH_1_ was strongly negatively correlated with both DL24 and DL48 (r = −0.77 and −0.68). Besides, meat color traits showed stronger genetic correlations with DL24 (r = 0.24 to 0.36) than with DL48 (r = 0.04 to 0.17). pH_1_ was strongly correlated with b* (r = −0.63), moderately with L* (r = −0.39), and weakly with a* (r = 0.07). Among meat color indicators, a strong positive correlation (r = 0.80) was observed between L* and b*. In contrast, L* and a* exhibited a moderate negative correlation (r = −0.60), as did a* and b* (r = −0.40).

## 4. Discussion

Phenotypic value distributions exhibited broad ranges for carcass and meat quality traits. The backfat thickness indicators, IMF, and drip loss indicators had higher coefficients of variation (CV > 50% for drip loss indicators) and lower homogeneity, suggesting greater breeding potential for these traits. Per the national agricultural industry standard of China (NY/T821-2019) [19], normal meat ranges are L* (37 to 52), pH_1_ (5.9 to 6.5), and DL48 (1.5% to 5.0%). Most Yorkshire pigs in this study exhibited normal meat characteristics based on the L*, pH_1_, and DL48. The IMF was 2.08% ± 0.59%, closely approximated to the results by Li et al. [20] (2.10% ± 0.89%) but higher than reports by Knapp et al. [21] (1.16% ± 0.44%) and Zhang et al. [22] (1.33% ± 0.08%) in Yorkshire pigs. Drip loss at 24 h and 48 h post-slaughter yielded 1.79% ± 1.30% and 3.94% ± 2.04%, respectively. Knapp et al. [21] (3.5% ± 2.4%) and Zhang et al. [22] (2.41% ± 0.26%) reported higher 24-h drip loss than our study, demonstrating inferior water retention capacity in their Yorkshire pigs. Knapp et al. [21] documented a pH_1_ of 6.35 ± 0.26, and Zhang et al. [22] obtained a pH_1_ of 6.81 ± 0.12 in Yorkshire pigs, both exceeding the pH_1_ observed in our study. Batch effects significantly influenced only a subset of carcass traits, likely due to similar farm and slaughter conditions across all five batches. Evaluator effects were significant for most carcass traits, highlighting the need to account for inter-operator variability in measurement. Sex significantly influenced most carcass traits, likely reflecting physiological differences in gender. Besides, batch effects only significantly influenced pH_1_ and b* in meat quality traits.

Although the Shapiro-Wilk test indicated deviation from a normal distribution for some traits, the GREML analyses, which were used to estimate heritability and genetic correlations, remained robust given the properties of linear mixed models (LMMs). LMMs are generally resilient to moderate departures from normality, particularly with large sample sizes [23]. The heritability of carcass length indicators was estimated at a mean of 0.35 in this study, while using pedigree information, Dube et al. [24] estimated it to be 0.33 in Yorkshire pigs, and Nakano et al. [25] estimated it to be 0.56 in Duroc pigs. Regarding the number of rib pairs, Borchers et al. [26] estimated a heritability of 0.51 in a Pietrain cross. Son et al. [27] estimated heritabilities of 0.24 and 0.78 in Landrace and Duroc pigs using significant SNP in GWAS. Typically, the number of ribs corresponds to the number of thoracic vertebrae. Thus, the heritability of thoracic vertebrae count estimated by Nakano et al. [25] (0.77) aligned with Son et al. [27] (0.78) in Duroc pigs. Based on the above results, we concluded that the heritability of NR varied widely among breeds. Backfat thickness heritability estimates ranged from 0.42 to 0.47 across studies: Dube et al. [24] reported 0.45 using pedigree information in Yorkshire pigs; Bergamaschi et al. [28] reported 0.415 using pedigree and genomic information in Duroc pigs; and Willson et al. [29] reported 0.38 using pedigree information in Duroc pigs. In this study, the heritability values were 0.33 for BFA, 0.29 for BFB, and 0.46 for BFC. These variations likely reflected differences in pig breeds and measurement methods. The heritability of EMA ranged from 0.22 to 0.55 [24,25,29,30], and our study’s estimate (0.28) was within this range.

The heritability of pH_1_ was estimated at 0.12 in this study. Among Yorkshire, Landrace, and Pietrain pigs, Knapp et al. [21] reported pH_1_ heritabilities of 0.19, 0.14, and 0.37, respectively, using pedigree information. Furthermore, heritability estimates for pH at 24 h post-slaughter was 0.15 for Miar et al. [30] in commercial crossbred pigs (Duroc × Landrace × Yorkshire), and was 0.08 for Khanal et al. [31] in two large commercial crossbred swine populations. Xie et al. [32] revealed that 698 Yorkshire pigs had moderate heritability (h^2^ = 0.29) for pH at 24 h post-slaughter based on genomic information. These findings collectively indicated that (1) pH generally demonstrated low heritability across porcine populations and that (2) heritability is breed specific. Similar to our study, Knapp et al. [21] estimated heritability for DL24 in Yorkshire, Landrace, and Pietrain pigs as 0.21, 0.10, and 0.10. The heritabilities of L*, a*, and b* in the present study generally agree with Xie et al. [32] in Yorkshire pigs. Knapp et al. [21] reported lower heritability (h^2^ = 0.26) estimates for meat color in Yorkshire pigs. IMF displayed high heritability (h^2^ > 0.4), aligning with reports by Khanal et al. [31] in two commercial crossbred populations and by Lo et al. [33] in a combined population (Landrace and Duroc).

Phenotypic correlations between carcass length indicators and backfat thickness indicators ranged from 0.02 to 0.16, whereas the genetic correlations showed negative associations (r = −0.91 to −0.20). Phenotypic correlations reflect the combined effects of genetics and environment. However, genetic correlations reveal the intrinsic biological relationship between traits. Strong negative genetic correlations indicate that genes promoting longer carcasses tend to reduce backfat thickness, possibly due to pleiotropy. Weak positive correlations suggest that environmental factors may simultaneously promote both carcass length and backfat thickness. Genetic correlations in the present study agreed with Nakano et al. [25], who reported negative phenotypic (r = −0.43) and genetic (r = −0.53) correlations in Duroc pigs. Contrastingly, Li et al. [1], applying GCTA software, identified positive phenotypic (r = 0.15) and genetic (r = 0.52) correlations in Yunong-black pigs. Similarly, Miar et al. [30] observed positive associations (phenotypic: r = 0.01; genetic: r = 0.19) in commercial crossbred pigs. Collectively, these results suggested that phenotypic and genetic correlations between carcass length and backfat thickness may vary in direction (negative or positive) depending on the population.

Phenotypic correlations between EMA and carcass length indicators ranged from 0.17 to 0.21, with genetic correlations spanning −0.17 to 0.23. Contrastingly, Miar et al. [30] reported stronger phenotypic (r = 0.35) and genetic (r = 0.47) correlations in commercial crossbred pigs, while Li et al. [1] observed correlations of 0.16 (phenotypic) and 0.40 (genetic) in Yunong-black pigs. The correlations between EMA and backfat thickness indicators showed weak phenotypic (r = −0.04 to 0.07) and genetic (r = −0.11 to 0.06) associations, particularly negative correlations with BFC. Similarly, Dube et al. [24] (phenotypic: r = −0.27; genetic: r = −0.41) and Miar et al. [30] (phenotypic: r = −0.38; genetic: r = −0.24) demonstrated inverse EMA and backfat thickness relationships.

Phenotypic correlations between NR and carcass length indicators ranged from −0.15 to 0.16, while genetic correlations showed strong positive associations (r = 0.44 to 0.85). The NR shows positive phenotypic and genetic correlations with nearly all carcass length indicators, indicating a consistent association between these carcass length indicators. The only exception was the phenotypic correlation between the NR and CL4, which was slightly negative. This isolated negative phenotypic correlation may be due to measurement-related factors influencing CL4 specifically. In contrast, the genetic correlations remained strongly positive across all indicators, suggesting a true biological positive association between rib number and overall carcass length. These findings align with Nakano et al. [25], who reported similar strong positive genetic correlations (r = 0.56) between thoracic vertebrae count and carcass length in Duroc pigs. Notably, NR exhibited negative phenotypic (r = −0.32 to −0.19) and genetic (r = −0.29 to −0.15) correlations with backfat thickness indicators. This inverse relationship was consistent with Nakano et al. [25], who documented a phenotypic correlation of −0.06 and genetic correlation of −0.16 between thoracic vertebrae number and backfat thickness.

Backfat thickness is a critical index in breeding programs. Synthesis of these findings reveals negative genetic correlations between backfat thickness indicators and both carcass length indicators and NR. Thiengpimol et al. [34] demonstrated that backfat thickness in 28-week-old primiparous Landrace sows reflected body condition and energy reserves and had a positive genetic correlation with reproductive performance. Hoa et al. [4] established that backfat thickness was an important index of meat quality in three-way crossbred pigs, with longissimus dorsi muscles demonstrating significantly superior flavor and juiciness in thicker backfat (21–30 mm) compared to thinner backfat (12–20 mm). Thus, selection for increased carcass length and NR as meat yield indicators risks unintended backfat thickness reductions, potentially compromising both sow reproductive performance and pork palatability. However, reduced backfat thickness may increase lean meat percentage. Therefore, it is essential to balance the economic importance of these traits and construct a comprehensive selection index. Water-holding capacity is important for meat quality and is difficult to measure [35]. Thus, Sevón-Aimonen et al. [35] used meat color indicators and pH at 24 h post-slaughter to predict drip loss of semimembranosus in two breeds (Landrace and Yorkshire) and showed that pH was the best predictor. Our findings confirm this pattern, as pH_1_ exhibited stronger phenotypic and genetic correlations with drip loss indicators than the meat color indicators.

## 5. Conclusions

After incorporating significantly fixed effects into the genetic parameter estimation model, we found that most of the carcass traits and meat quality traits of Yorkshire pigs were of medium to high heritability, and only pH_1_ showed low heritability. The results of phenotypic and genetic correlation analyses of two traits in the same population varied. Carcass length indicators had high genetic correlations with backfat thickness indicators and the NR. Drip loss indicators had strong negative genetic correlations with pH_1_.

## Figures and Tables

**Table 1:** The nomenclatures, definitions, and abbreviations of the carcass and meat quality traits.

Class of Traits	Nomenclature	Definition	Abbreviation
Carcass traits	Thoracolumbar length	Length from the first thoracic vertebra to the last lumbar vertebra	CL1
	Carcass length	Length from the anterior edge of the pubic symphysis to the anterior edge of the first cervical spine	CL2
	Carcass straight length	Length from the lumbosacral junction to the anterior edge of the first cervical spine	CL3
	Carcass slanting length	Length from the anterior edge of the pubic symphysis to the junction of the first rib and sternum	CL4
	Backfat A	Backfat thickness in the withers	BFA
	Backfat B	Backfat thickness in the thoracolumbar junction	BFB
	Backfat C	Backfat thickness in the lumbar spine junction	BFC
	Number of rib pairs	Number of rib pairs	NR
	Eye muscle area	Area of cross section of longissimus dorsi between the 3rd and 4th last ribs	EMA
Meat quality traits	Intramuscular fat content	-	IMF
	Drip loss at 24 h	-	DL24
	Drip loss at 48 h	-	DL48
	pH value measured within one hour after slaughter	-	pH_1_
	Meat color values of lightness, redness, and yellowness within 45 to 60 min after slaughter	-	L*, a* and b*, respectively

**Table 2:** Descriptive statistics and normality test for carcass traits.

Trait	Statistic	Mean Value	Standard Deviation	Coefficient of Variation (%)	Minimum Value	Maximum Value	Shapiro-Wilk
CL1 (cm)	461	72.76	3.36	4.61	60.00	85.00	0.0023
CL2 (cm)	461	102.87	3.41	3.31	92.00	114.00	0.1611
CL3 (cm)	461	87.45	3.39	3.87	73.00	100.00	0.0006
CL4 (cm)	461	88.84	3.63	4.09	77.00	100.00	0.2970
BFA (mm)	461	39.38	7.32	18.60	13.31	62.13	0.2248
BFB (mm)	461	24.58	5.57	22.65	9.32	47.72	0.0771
BFC (mm)	461	16.62	4.49	27.04	5.80	34.10	0.0009
EMA (cm^2^)	461	49.03	5.76	11.74	32.30	69.20	0.0413
NR (pairs)	461	14.30	0.81	5.67	13.00	17.00	<2.2 × 10^−6^

**Table 3:** Descriptive statistics and normality test for meat quality traits.

Trait	Statistic	Mean Value	Standard Deviation	Coefficient of Variation (%)	Minimum Value	Maximum Value	Shapiro-Wilk
IMF (%)	459	2.08	0.59	28.27	1.00	4.99	6.16 × 10^−12^
DL24 (%)	461	1.79	1.30	72.75	0.11	9.20	<2.2 × 10^−16^
DL48 (%)	461	3.94	2.04	51.67	0.23	14.83	6.96 × 10^−14^
pH_1_	461	6.02	0.33	5.44	4.95	6.74	1.48 × 10^−9^
L*	460	46.26	2.08	4.50	40.77	53.12	3.07 × 10^−5^
a*	460	16.44	1.16	7.04	13.15	19.98	0.3873
b*	460	4.38	0.81	18.39	2.36	7.33	4.27 × 10^−6^

**Table 4:** Fixed-effects significance test for carcass and meat quality traits.

Class of Traits	Trait	Batch	Sex	Evaluator
Carcass traits	CL1		**	***
	CL2			**
	CL3	*	*	***
	CL4			**
	BFA		**	**
	BFB		***	
	BFC	***	***	***
	EMA	***	***	-
	NR		*	***
Meat quality traits	IMF		***	-
	DL24		*	-
	DL48		***	-
	pH_1_	***	*	-
	L*		**	-
	a*			-
	b*	***		-

*** *p* < 0.001, ** *p* < 0.01, and * *p* < 0.05.

## Data Availability

The datasets used and analyzed during the current study are available from the corresponding author on reasonable request.

## Abbreviations

The following abbreviations are used in this manuscript:

DFD	Dark, firm, and dry
PSE	Pale, soft, and exudative
CL1	Thoracolumbar length
CL2	Carcass length
CL3	Carcass straight length
CL4	Carcass slanting length
BFA	Backfat A
BFB	Backfat B
BFC	Backfat C
NR	Number of rib pairs
EMA	Eye muscle area
IMF	Intramuscular fat content
DL24	Drip loss at 24 h
DL48	Drip loss at 48 h
pH_1_	pH value measured within one hour after slaughter
L*	Meat color values of lightness within 45 to 60 min after slaughter
a*	Meat color values of redness within 45 to 60 min after slaughter
b*	Meat color values of yellowness within 45 to 60 min after slaughter
GREML	Genome-based Restricted Maximum Likelihood
